# Macrophages in aseptic loosening: Characteristics, functions, and mechanisms

**DOI:** 10.3389/fimmu.2023.1122057

**Published:** 2023-03-08

**Authors:** Yehao Cong, Yi Wang, Tao Yuan, Zheng Zhang, Jianxun Ge, Qi Meng, Ziqing Li, Shui Sun

**Affiliations:** ^1^ Department of Joint Surgery, Shandong Provincial Hospital Affiliated to Shandong First Medical University, Jinan, Shandong, China; ^2^ Orthopaedic Research Laboratory, Medical Science and Technology Innovation Center, Shandong First Medical University & Shandong Academy of Medical Sciences, Jinan, Shandong, China; ^3^ Department of Joint Surgery, Shandong Provincial Hospital, Shandong University, Jinan, Shandong, China

**Keywords:** macrophage, polarization, aseptic loosening, arthroplasty, inflammation

## Abstract

Aseptic loosening (AL) is the most common complication of total joint arthroplasty (TJA). Both local inflammatory response and subsequent osteolysis around the prosthesis are the fundamental causes of disease pathology. As the earliest change of cell behavior, polarizations of macrophages play an essential role in the pathogenesis of AL, including regulating inflammatory responses and related pathological bone remodeling. The direction of macrophage polarization is closely dependent on the microenvironment of the periprosthetic tissue. When the classically activated macrophages (M1) are characterized by the augmented ability to produce proinflammatory cytokines, the primary functions of alternatively activated macrophages (M2) are related to inflammatory relief and tissue repair. Yet, both M1 macrophages and M2 macrophages are involved in the occurrence and development of AL, and a comprehensive understanding of polarized behaviors and inducing factors would help in identifying specific therapies. In recent years, studies have witnessed novel discoveries regarding the role of macrophages in AL pathology, the shifts between polarized phenotype during disease progression, as well as local mediators and signaling pathways responsible for regulations in macrophages and subsequent osteoclasts (OCs). In this review, we summarize recent progress on macrophage polarization and related mechanisms during the development of AL and discuss new findings and concepts in the context of existing work.

## 1 Introduction

Total joint arthroplasty (TJA) is an extensive and successful surgical therapy, which has been used in the treatment of severely traumatic or arthritic joint diseases. It helps in relieving arthralgia, rebuilding locomotor function, and improving living quality (1). Even so, the long-term survival of joint prosthesis reduces over time, which leads to implant failure, reduced locomotor ability, and heavy financial burden.

The main reason for the failure of TJA is that the joint interface continuously produces debris particles which induce a complex inflammatory response and leads to osteolysis and aseptic loosening (AL) (2–4). At present, the major pathogenesis of AL is the production of wear debris due to the mechanical strength of joints and biological interactions over time. On one hand, the debris induces functional changes in macrophages and promotes the release of cytokines to regulate the immune microenvironment in the bone-implant interface. On the other hand, by further affecting the bone remodeling process, the debris increases bone resorption, eventually resulting in AL (3–6).

Macrophages, the major population of tissue-resident mononuclear phagocytes, are a critical class of cells that have an effect on bacterial recognition and elimination, as well as in the process of innate and adaptive immunity (7). Macrophages have a variety of functions, exhibiting different phenotypes based on practical conditions and responding to microenvironmental signals. Two major phenotypes of macrophages include classically activated macrophages (M1) and alternatively activated macrophages (M2) (8–10). M1 macrophages have proinflammatory properties and are involved in initiating and maintaining the inflammatory state, whereas M2 macrophages have anti-inflammatory properties and take part in tissue homeostasis and repair (8–13).

During the development of inflammation, the polarized state of macrophage is in a dynamic equilibrium. In this regard, macrophages can distinctively adapt to the microenvironment, respectively (9). For example, the increased count of proinflammatory M1 macrophages induced by pathological stimulus leads to periprosthetic osteolysis, whereas anti-inflammatory M2 is favorable to shape an immunomodulatory environment towards osseointegration (14, 15). Therefore, it is essential to figure out the polarization of macrophages and associated regulatory mechanisms during the pathogenesis of AL.

At present, the understanding of macrophages is still limited to some extent. For the past few years, advancing awareness of the impact of macrophage polarization on the pathogenesis of AL has been recognized. This article reviewed interactions between the various receptors, ligands, signal transductions, and other factors related to functional changes of macrophage around the prosthesis. In addition, it cited the research conclusions and reviews regarding other macrophage-related inflammatory regulations, and also emphasized the importance of the functional changes and regulatory mechanisms of macrophages in the bone-implant interface microenvironment.

## 2 Overview of aseptic loosening

### 2.1 Clinical features

Harris et al. (16) described a phenomenon of extensive bone resorption leading to loosening without infection in four patients after receiving hip arthroplasty surgery, and this is the first detailed description of AL (16). In definition, AL can be generally described as a failure of the fixation of one or more prosthetic components without any infection (17). It may probably originate from inadequate initial fixation, mechanical loss of fixation over time, or biological loss of fixation, all of which are the leading causes of particles-induced osteolysis around the prosthesis (4, 17). Joint pain is the typical symptom of patients with AL, which always become worse when the affected joint carries out physical activity or bears weight. Impaired gait and restricted range of motion are often discovered in the physical exam of these patients (17).

As one of the major reasons for the failure of artificial joint implants, AL is the main cause of revision surgery (18–20). Due to the high complication rate, the requirement for complex technology, and the heavy economic burden brought by revision surgery (18), extensive studies have focused on the pathogenesis of AL in order to develop diagnostic and therapeutic avenues with more sensitivity and efficacy. The majority of published works reported that AL is mainly caused by wear particles-induced periprosthetic osteolysis (PPOL) (2–4).

### 2.2 Pathogenesis

Loosening of the prosthesis is a very complex process, involving many mechanical and biological aspects (21, 22). The main biological factor is the biological response of cells to a variety of wear particles (21), for example, wear particles can promote macrophage polarization to M1, and release proinflammatory cytokines and chemokines (23, 24). These cellular responses and subsequent activities are determined by many factors, such as the physical and chemical properties of the material, including the size, morphology, and composition of the material (23, 25–29). Moreover, the presence of endotoxin can also affect these cellular responses and activities (30–32). From the aspects of the disease host, patient-related risk factors, such as age, sex, obesity, smoking, and genetic variation, also play a role in AL pathogenesis (33–38). However, comorbidities affect the occurrence of AL even larger. Patients with hemophilia are reported to have a higher risk of AL (39). Elevated inflammatory activity will increase the risk of loosening after TJA in patients with rheumatoid arthritis (RA), thus the indication of arthroplasty for RA patients should be more strictly controlled (40).

### 2.3 Pathological feature

The chronic inflammation at the bone-implant interface, accompanied by osteolytic destruction in the surrounding bone, is the major pathological feature of AL (5, 6, 41, 42). Based on histopathological findings, there are numerous infiltrating CD68-positive mononuclear/macrophages, foreign body giant cells (sometimes organized as foreign body granulomas), and wear particles in the periprosthetic connective tissue (42, 43). Scattered fibroblasts and T cells can be observed in the surrounding area of infiltrating macrophage (42). In addition, endotoxin contamination is also present around the prosthesis (44, 45). Regarding the cytokines, there is a significant increase in the expression of proinflammatory factors in the tissue, including interleukin-1β (IL-1β), IL-2, IL-8, interferon-γ (IFN-γ), and tumor necrosis factor-α (TNF-α) (41). It was found that in the mice skull implanted with titanium (Ti) particles, macrophages polarized into M1 macrophages in the early phase of the inflammatory responses, and partial tissue restoration was observed in the resolution of inflammation after 6 to 8 weeks (46). However, long-term chronic inflammation eventually leads to osteolytic destruction (6). Studies on the various polarization phenotypes of macrophages may help in further explaining the pathogenesis of AL.

## 3 Overview of macrophages in aseptic loosening

### 3.1 Origin of macrophages and wear particles

Monocytes/macrophages originally come from the hematopoietic stem cell (HSC) in the bone marrow and subsequently enter the peripheral blood. In responding to the local inflammation, circulating monocytes leave the bloodstream and mobilize into the local tissues. Upon stimulation by several growth factors, proinflammatory cytokines, or microbial products, circulating monocytes further differentiate into macrophages (11, 12). In addition to that, resident tissue macrophages recruited from the bone marrow are necessary drivers of inflammatory and tissue regenerative responses (47). The initial recruitment of inflammatory cells results from chemotactic factors produced by macrophages (48). When circulating macrophage or monocyte recruitment or activation is disrupted, the early inflammatory response is often diminished (49). Conclusively, the number of tissue-resident macrophages can increase exponentially, including locally proliferating macrophages and monocytes recruited from the bone marrow (50–52).

Due to the detection of ultra-high molecular weight polyethylene (UHMWPE) and various kinds of high-density material debris in tissue samples from patients, submicron-sized wear particles are usually considered potential causes of AL pathogenesis (53). During disease progress, macrophages play a crucial role in recognizing wear particles and releasing a large number of proinflammatory cytokines and chemokines, such as IL-1β, TNF-α, IL-6 (15, 23, 24, 26, 54–58). Diverse cytokines individually modulate the function of cells located at the interface between the prosthesis and the surrounding bone, and collectively affect other cells through diverse signaling mechanisms, ultimately leading to particles-induced inflammatory osteolysis. Detailed interactions between cytokines and cells are to be reviewed in section 4.

### 3.2 PAMPs and subclinical infection

Common pathogen-associated molecular patterns (PAMPs) include lipopolysaccharide (LPS) and lipoteichoic acid (LTA). LPS is a typical endotoxin and a major component of the outer cell wall of Gram-negative bacteria, whereas LTA is a cell wall polymer discovered in Gram-positive bacteria (59, 60). PAMPs regulate macrophage polarization through toll-like receptors (TLRs) and further promote the release of cytokines (61–63).

Previous studies have reported that some active bacteria or its structural components could be found in the tissues surrounding the loosened implant, even in the absence of any clinical or microbial evidence of infection (44, 45). In fact, subclinical infection is difficult to identify, and the probable cause of this is the growth pattern of biofilm. By firmly anchoring to the surface of the implanted prosthesis, biofilm may have protected inside microorganisms that are being infected from elimination, therefore it is reasonable to suspect these bacterial biofilms anchoring on the surface of the loosened implant as the latent source of endotoxin (45, 64). In addition, PAMPs may also originate from bacterial colonies residing in the gastrointestinal tract, the oral cavity, or even the wounds in the skin, where these bacteria and PAMPs are occasionally transferred to the circulating blood, in turn reaching the implant (65, 66). Evidenced by an *in vivo* experiment based on mouse balloon models, endotoxin in blood circulation could adhere to Ti particles and consequently induce macrophage aggregation (67).

### 3.3 Functional changes of macrophages

**Figure 1:**
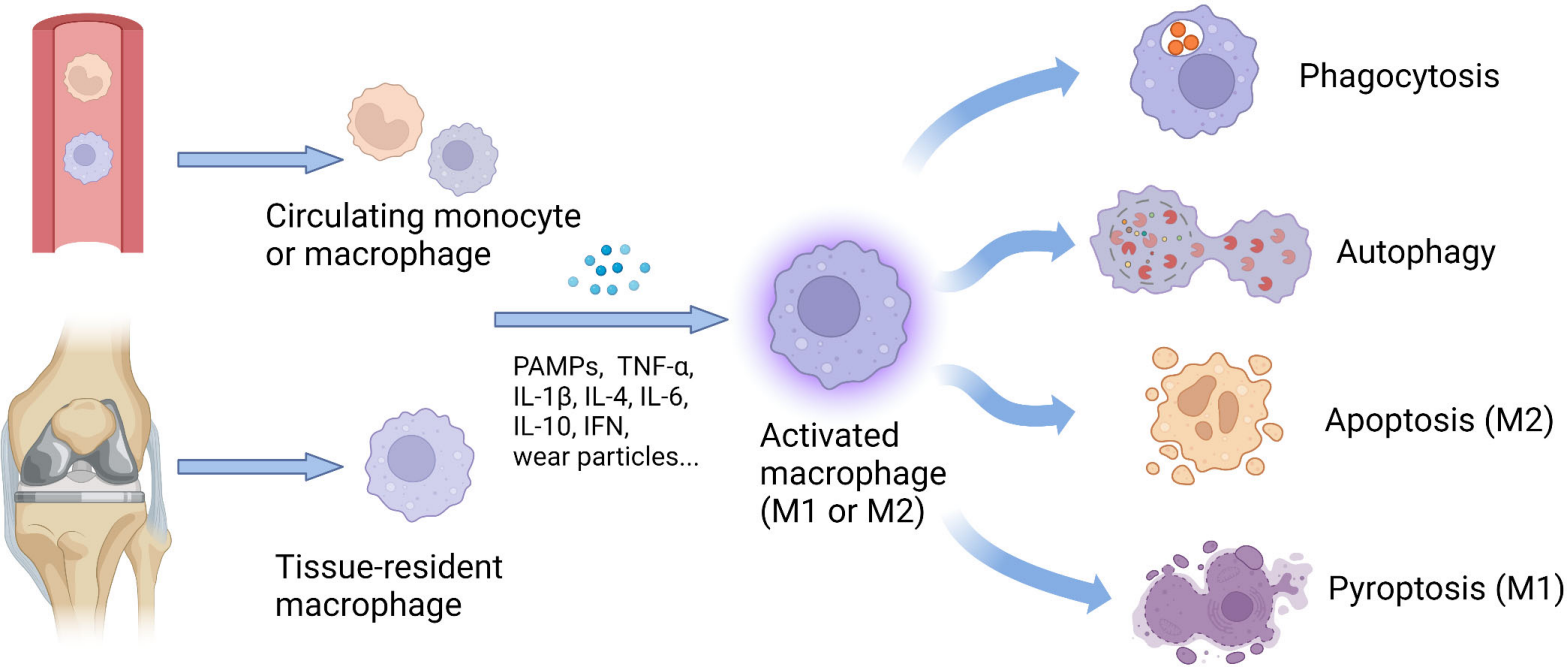
Origin and functional changes of macrophages. The number of tissue-resident macrophages can increase exponentially, including locally proliferating macrophages and monocytes recruited from the circulating peripheral blood. Stimulated by various factors, such as cytokines, PAMPs, and wear particles, the macrophages with different origins could be activated and further polarize into M1 or M2 phenotype. According to cellular phenotypes, activated macrophages show diverse functions, including phagocytosis, autophagy, apoptosis, and pyroptosis, to defend against infections and present antigens to other immune cells, thereby regulating the immune responses. IFN, interferon; IL, interleukin; M1, classically activated macrophages; M2, alternatively activated macrophages; PAMPs, pathogen-associated molecular patterns; TNF-α, tumor necrosis factor-alpha.

Macrophages are activated and alter their functions to defend against infections and present antigens to other immune cells, thereby regulating the immune responses (**Figure 1**) (68).

#### 3.3.1 Proinflammatory and anti-inflammatory functions

Macrophages amplify the inflammatory process by releasing proinflammatory factors to remove pathogens or other foreign bodies (9, 47). During tissue injury, the local cells which are infected by pathogens and undergo necrosis or pyroptosis could release PAMPs or damage-associated molecular patterns (DAMPs) that activate inflammatory signaling in macrophages and other resident cell populations. Activated cells recruit neutrophils, monocytes, and other inflammation-related cells into the tissue by a release series of cytokines. Once the acute injury is under control, macrophages supply nutrition to the tissues where they are located by decomposing remnants and secreting growth factors and mediators, exerting their function effectively in inhibiting inflammation (47). Some macrophages, characterized by producing of growth factors, including platelet-derived growth factor (PDGF), insulin-like growth factor 1 (IGF-1), and vascular endothelial growth factor-α (VEGF-α), are associated with tissue repair and help in promoting cell proliferation and vascular development and thus alleviating local hypoxia that occurs after injury (47). They also produce soluble mediators, such as transforming growth factor-β1 (TGF-β1) that induce local and recruited fibroblasts to differentiate into myofibroblasts, thereby synthesizing extracellular matrix components and promoting wound closure (69). In the final phase of tissue repairment, monocytes and macrophages present an anti-inflammatory phenotype (47, 70). It was found that these macrophages responded to inhibitory mediators such as IL-10 in the local microenvironment, eventually leading to relieving inflammation (71, 72).

Phagocytosis mainly belongs to the function of M1 macrophages, which goes along with the proinflammatory process, although M2 macrophages also demonstrate a weak function in phagocytosis (73, 74). Stimulated by the serum from Behçet’s disease (BD) patients, monocyte-derived macrophages (MDMs) could differentiate into M1 macrophages with enhanced phagocytic capacity (75). Subsequently, M1 macrophages display an enhanced capacity for the elimination of pathogens, which largely results from their increased production of superoxide, NO, and their derivatives (76). M2 MDMs also demonstrate the ability to phagocytose escherichia coli and cancer cells. Further research found that the phagocytosis in M2 macrophages mainly owes to their surface markers, such as CD14, CD206, and CD163 (77). However, compared with M0 and M2 macrophages, LPS-treated M1 macrophages exhibited an obviously higher ability for phagocytic activity (78).

#### 3.3.2 Autophagy

Both autophagy and phagocytosis in macrophages are lysosomal-dependent catabolic processes, by which cells can engulf and deliver cargo to the lysosomes for digestion *via* forming transient vesicular structures (autophagosome and phagosome) (79). Acting as scavenger cells, macrophages could phagocytize cellular debris, invading pathogens, and other apoptotic cells (80, 81). Phagocytizing dead cells in macrophages contribute to diverse immune and inflammatory signals that could also trigger intracellular autophagy in macrophages (82, 83). Other studies have revealed more relations between autophagy and phagocytosis. Autophagy promotes phagocytosis and the clearance of pathogens *via* the NOD-like receptor family pyrin domain containing 3 (NLRP3) inflammasome in macrophages (84). As a novel function for autophagy proteins, the LC3-associated phagocytosis pathway (LAP) is closely associated with phagocytosis in macrophages, which exerts its role in blocking proinflammatory signals upon phagocytosis of dying cells and preventing the presentation of autoantigen to other cells (85, 86). Interestingly, autophagy-deficient macrophages may boost phagocytosis through increased scavenger receptor expression (87).

Regarding the functional changes in activated macrophages, vitamin D could restore anti-inflammatory M2 macrophages in an autophagy-dependent manner (88). Similarly, ubiquitin-specific protease 19 (USP19) could inhibit inflammatory responses and promote M2 polarization by increasing autophagy flux (89). Controversially, another study reported that inhibition of autophagy could drive macrophages to the M2 phenotype (90). In AL, a recent study revealed that by activating LAP, aluminum oxide nanoscale particles (Al-n) attenuated the macrophage M1 polarization and inhibited the secretion of inflammatory factors, leading to the prevention of the AL pathogenesis induced by particles *in vivo* (26). Although autophagy has been shown to be involved in the regulation of macrophage polarization, evidence regarding the regulatory mechanisms is underdeveloped.

#### 3.3.3 Apoptosis

Apoptosis is the sequential death of cells *via* the mechanisms called programmed cell death (PCD) (91), which is characterized by morphological changes in the cellular structures together with a series of enzyme-dependent biochemical processing (92). In the resolution phase of inflammation, the infiltrating leukocytes execute the acute innate response and undergo apoptosis, subsequently cleared by phagocytic macrophages. In this course, macrophages undergo reprogramming from inflammatory to anti-inflammatory, leading to the relief of inflammation (93).

Efficient clearance of early apoptotic cells requires macrophages polarizing into the M2c phenotype (94). The capacity of M2 macrophages to uptake apoptotic cells depends on several necessary molecules, such as Mer tyrosine kinase, Axl receptor tyrosine kinase, growth arrest-specific 6 (Gas-6) (94, 95). In addition, the macrophage itself could also undergo apoptosis induced by wear particles (96). Consequently, the increase in macrophage apoptosis limits the proinflammatory function of macrophages (97, 98), seemly to be another important mechanism regarding the delayed inflammatory response in AL. The caspase-3, a key mediator related to apoptosis, was detected in periprosthetic tissues in a mouse osteolysis model induced by UHMWPE particles (99, 100). Furthermore, apoptosis is associated with phenotypic changes in macrophages. Transcription factor Zhx2 deficiency could enrich the expression of M2 phenotype markers and as well promote the apoptosis of macrophages (101). Likewise, through the inhibition of M2-specific gene expression and apoptotic cell death, Delta-like-ligand 4 (DLL4) may prevent macrophage from polarizing into the M2 phenotype (102).

#### 3.3.4 Pyroptosis

Different from apoptosis, pyroptosis is a proinflammatory type of PCD mediated by the gasdermin family, which usually leads to cell swelling and rapid rupture of plasma membranes, as well as the release of immunogenic cell contents, thereby exaggerating inflammatory status (103). In the aspect of the immune response, pyroptosis induced by the activation of pattern recognition receptors (PRRs) can stimulate inflammatory responses, independent of its effect in promoting cytokine induction (104). Pyroptosis is undoubtedly related to macrophage polarization. On one hand, by secreting exosomal cathepsin S, M1 macrophages induce pyroptosis in pancreatic acinar cells *via* the caspase1-mediated classical pyrolysis pathway, resulting in inflammation and pancreatic tissue damage (105). On the other hand, exosomal Mir-30D-5p of polymorphonuclear neutrophils (PMNs) is reported to induce M1 polarization by upregulating TNF-α, IL-1β, IL-6 and triggering pyroptosis in macrophages, leading to sepsis-associated acute lung injury (106).

Moreover, some mutual signals could mediate both M1 polarization and pyroptosis in macrophage, such as the caspase-1/GSDMD signaling pathway (107) and METTL3/MALAT1/PTBP1/USP8/TAK1 axis (108). The overexpression of brain and muscle Arnt-like protein 1 (BMAL1) also reduces the production of inflammasomes and pyroptosis in macrophages, as well as decreases the proportion of M1 phenotype *via* the TLR2/NF-κB pathway (109). A recent study has found that macrophages can activate the NLRP3 inflammasome and initiate subsequent pyroptosis to affect AL pathogenesis in mice model of cobalt-chromium-molybdenum (CoCrMo) alloy particles-induced osteolysis (110). As a result, wear particles not only induce M1 polarization and production of proinflammatory cytokines, but also boost inflammation by increasing pyroptosis in macrophages and inducing local tissue impairment.

### 3.4 Bone remodeling and osteoclastogenesis

Bone remodeling is a dynamic and balanced process maintained by osteoblasts (OBs) and osteoclasts (OCs), which is deeply affected by the receptor activator of nuclear factor NF-κB ligand/osteoprotegerin (RANKL/OPG) ratio. Disruption of this homeostasis leads to severe skeletal disorders (111). OBs are a type of bone-forming cells that are derived from bone marrow mesenchymal stem cells (BMSCs) and could respond to anabolic factors, such as bone morphogenic proteins (BMPs) (112). On the other hand, OCs are the unique cells known to absorb bone at or near the bone surface, which originate from bone marrow-derived monocytes/macrophages (BMMs) (113, 114).

Osteoclastogenesis is a complex process involving events of proliferation, differentiation, cell fusion, and multinucleation (111). The primary osteoclastogenic factor is RANKL which triggers a complex network of signaling pathways including NF-κB and mitogen-activated protein kinases (MAPK), *via* the receptor RANK on OC progenitors. By further activating the nuclear factor of activated T cells c1 (NFATc1), the master transcriptional factor of osteoclastogenesis, the fate of OC progenitors is decided by the controlling of key osteoclastogenic genes, such as tartrate-resistant acid phosphatase (TRAP) and cathepsin K (CTSK) (113–115). Calcineurin, a powerful mediator of transcriptional activity of NFATc1, is regulated by cytosolic calcium (Ca^2+^) downstream of the TEC kinases and phospholipase Cγ (PLCγ), all of which are governed by both RANK and immunoreceptor tyrosine-based activation motif (ITAM)-based signaling. In addition, sustained intracellular Ca^2+^ oscillations are required for OC formation and functions, which will be introduced in the following section 5.5.3 (116). On the opposite side, OPG, as a decoy receptor, blocks RANKL binding to its cellular receptor RANK. An active OC is a highly polarized cell with a distinctive cytoskeletal organization and has the ability to create the sealing zone which is a site of tight membrane apposition to the bone surface, thereby executing its function as a bone-resorbing machine (116).

## 4 Polarization and phenotype of macrophages

Two major phenotypes of macrophages in response to environmental stimuli are M1 and M2. Usually, the phenotype of macrophages can be transformed by reprogramming (117, 118). Moreover, a recent study based on a spectrum of activated human macrophages revealed that there are continuous intermediate phenotypes between two opposite terminal phenotypes (119). Under such background, researchers use the term “polarization” to define the preference pattern of gene expression and protein synthesis in macrophages after different stimuli (120). “Naive” M0 macrophages, the prototype of M1 and M2 macrophages, are characterized by the expression of CD11b and F4/80, and emerge from committed myeloid progenitors in the presence of macrophage colony-stimulating factor (M-CSF). Although lacking the expression of antigen-presenting molecules (MHC-II) and co-stimulatory molecules (B7), M0 macrophages could readily phagocytose cellular debris or pathogens (78, 121).

The polarization to M1 macrophages has two main inducible sources, either microbial products or the cytokines secreted by TH1 lymphocytes. By recognizing pathogens and presenting antigens to T lymphocytes, M1 macrophages play a critical role in triggering adaptive immunity in the body. During immunization, M1 macrophages produce high amounts of proinflammatory factors (IL-1 β, IL-6, TNF-α, IL-12, and IL-23, among others), which in turn promote Th1 proinflammatory response (8, 10, 12, 122, 123). In contrast, M2 macrophages are mainly responsible for inflammatory relief and tissular repairs. Discovered in the early 1990s, the polarization to M2 macrophages is related to IL-4, IL-10, or IL-13 which are produced by innate and adaptive immune cells, such as mast cells, basophils, and Th2 lymphocytes (9, 122–124). By producing multiple growth factors and cytokines, such as TGF-β, IGF-1, PDGF, VEGF, IL-8, and endothelial growth factor (EGF), M2 macrophages are aided in the suppression of local inflammation and thus beneficial for tissue repairs (8, 10, 12, 122).

### 4.1 M1 macrophage in AL

#### 4.1.1 Stimulus from wear particles and endotoxin

In the bone-implant interface, macrophage polarized to the M1 phenotype is mainly stimulated by wear particles and endotoxin (55). Upon continuous stimulation, M1 macrophages cause tissue damage by strengthening local inflammation *via* the secretion of TNF-α, IL-1β, IL-6, and IL-8 and the reduction of IL-10 expression (15, 23, 24, 26, 54–58). Besides, other cytokines such as IFN-β (15) and various chemokines, including chemokine (C-X-C motif) ligand 9 (CXCL9), CXCL10, and CXCL11 (56) secreted by M1 macrophages also play a role in AL pathogenesis. These biological reactions are largely affected by the size and type of wear particles (23, 26). Endotoxin also contributes to macrophage differentiation into the M1 phenotype by stimulating TLRs on the cell surface. Particles with PAMPs adhering, such as LTA and LPS, could induce more production of the proinflammatory cytokine than those without endotoxin (30, 32). On the contrary, the removal of endotoxins from particles significantly reduces the cellular activity of macrophages (30) and inhibits OC differentiation (31).

#### 4.1.2 Surface receptor and DAMPs

It is well-established that both polyethylene (PE) and Ti particles can promote the expression of TLRs and various proinflammatory factors in macrophages (43, 125). A recent *in vivo* experiment also confirmed that alloy particles could induce significantly higher numbers of TLR-1, -4, and -6 positive cells in the synovial layer of joints (126). In the downstream of TLRs, NLRP3, ASC, caspase-1, and TNF-α and IL-1β were found to be positive through the colocalization with CD68 in the tissues around the revised prosthesis (43). The NF-κB, MAPK, and TAK1 pathways are involved in mediating signaling transduction downstream of TLRs (61–63, 127–129). In addition to TLRs, macrophages can engulf PMMA debris through macrophage receptors with collagenous structure (MARCO), which is a key pathogenic factor in promoting the phagocytosis of polymethyl methacrylate (PMMA) debris in aging macrophages (130).

As the main alternative hypothesis to PAMPs, DAMPs also activate TLRs during AL. The term “DAMPs” refer to self-molecules released by dying or damaged cells, which are defined as endogenous danger molecules due to they could activate the innate immune system by interacting with pattern recognition receptors (PRRs) (131). DAMPs are recognized by various membrane-bound receptors, including PRRs and non-PRRs, and also by intracellular sensors, notably through inflammasome (132, 133). Through binding to specific receptors, DAMPs activate the inflammatory process and recruit immune cells like neutrophils and monocytes. Therefore, after the clearance of DAMPs, the recruited leukocytes will change from a proinflammatory into a reparative program (133). Cobalt alloy particles could induce macrophage-associated inflammatory responses and bone loss through DAMPs rather than activated TLR4, due to the partially absent of metal-binding histidines in TLR4 (134). However, it has also been thought that the DAMPs generated in response to particles are insufficient to activate TLR2 or TLR4 in these cells (32).

Although DAMPs contribute to the host’s immune defense, they also promote pathological inflammatory responses. The DAMPs, such as high-mobility group box 1 (HMGB1), S100 proteins, and heat shock proteins (HSPs), are commonly known as regulatory molecules of inflammatory responses (131). HMGB1 is an ancient DNA-binding nucleoprotein. It can be passively released from dying cells or actively secreted by monocytes, macrophages, and myeloid dendritic cells (135, 136). HMGB1 could induce M1 polarization *via* TLR2, TLR4, and RAGE/NF−κB signaling pathways, leading to LPS−induced acute lung injury (124), meanwhile, it significantly yielded the expression of the M1 marker inducible nitric oxide synthase (iNOS) while decreasing the M2 marker IL-10 in macrophages (137). *In vitro*, HMGB1 silencing down-regulated the secretion of inflammatory cytokines in macrophages, which cannot be reversed by the exogenous HMGB1 (138). Interestingly, HMGB1 could also trigger M2 macrophage polarization *via* the TLR2/NOX2/autophagy axis (139). In the same aspect, loss of HMGB1 in macrophages can increase the differentiation of proinflammatory macrophages and enhance inflammatory response under specific conditions, not the otherwise (140). In this regard, HMGB1 may induce distinct macrophage phenotypes probably due to different redox isoforms (141). Besides HMGB1, the expression level of several members of HSPs is closely related to distinct stages of polarization in macrophages (142). Compared with unpolarized macrophages, a significant up-regulation of members of the HSP70 family (HSPA2 and HSPA8), as well as the HSP90 family (HSP90AA1) can be observed in M1 macrophages. On the other hand, changes in HSP expression were also observed in macrophages during the M2 polarization, although with only five transcripts being significantly modulated. Among them, DNAJB5, HSPA13, HSPBAP1 were upregulated, whereas HSPH1 and HSPB1 were down-regulated (142).

#### 4.1.3 Particles-induced inflammation conduce to osteolysis

Recent studies have found that Ti, CoCrMo particles, and LPS could strongly induce inflammatory responses in macrophages, significantly increasing the production of TNF-α and IL-1β, rising RANKL/OPG ratio, and enhancing the OC activity (143–146). The current paradigm holds the view that the induction of OC differentiation by inflammatory cytokines is indirectly yielded under RANKL stimulation (147). Proinflammatory cytokines, such as IL-1β, TNF-α, IL-6, soluble IL-6 receptor, and IL-17, could all increase the production of RANKL from OBs. The effects of proinflammatory cytokines could be balanced by anti-inflammatory cytokines, such as IL-4 and IL-13, which could inhibit RANKL expression (147). In addition, a variety of wear particles could conduce to osteolysis *via* elevating M1 polarization (15, 24, 57, 58), since M1 macrophages are an important source of TNF-α production (11).

Although the concrete mechanism of TNF-α on OC differentiation is not fully understood, TNF-α signals promote OC differentiation by upregulating several proinflammatory target genes through the activation and nuclear translocation of NF-κB. One such target is RANK, which increases OC activity by mediating RANKL signaling (148). Interestingly, TNF-α produced by LPS/TLR4 signals can regulate OC generation in LPS-treated macrophages through the activation of RANKL signaling, whereas TNF-α in a RAW264.7 cells-based experiment demonstrates it may act as an autocrine/paracrine factor in promoting osteoclastogenesis, independent of RANKL signaling (149). In addition, the co-culture experiments of macrophages and MSCs (or OBs) revealed that PE and Ti particles could inhibit OB function, meanwhile promoting M1 polarization and osteoclastogenesis (150–152), proposing another mechanism for inflammation-induced osteolysis.

### 4.2 M2 macrophage in AL

#### 4.2.1 Subtypes of M2 macrophage

The classical M1/M2 system was based on experiments *in vitro* with different stimulation approaches. Subsequent studies revealed that the process of macrophage activation and polarization is much more complex and needed an additional subdivision of the M2 population (153). As a result, the M2 macrophages is further classified into four subtypes: alternative activated macrophages (M2a), type 2 macrophages (M2b), deactivated macrophages (M2c), and M2-like macrophages (M2d) (154). Among them, applied stimuli and the achieved transcriptional changes correspond to particular subtypes: 1) M2a macrophage induced by IL-4 or IL-13 is a profibrotic phenotype, 2) M2b macrophage is stimulated by immune complexes combined with Toll-like receptor or IL-1 receptor agonists, and 3) M2c macrophage is exposure to IL-10, TGF-β, or glucocorticoids, 4) M2d, activated by adenosines or IL-6 (154, 155). For the pathological study, although M2a/c macrophage is found to be beneficial in early inflammatory stages, they have been uncovered to impair tissue remodeling (156). Similarly, M2b macrophages have been thought to be relieving immune responses with minor damage to local tissue (157).

#### 4.2.2 M2 macrophage conduces to inflammatory relief

M2 macrophage has the effect of alleviating inflammation induced by wear particles, through increased expression of IL-10 and decreasing expression of IL-6 and TNF-α (24, 57, 58). In the presence of large amounts of TLR2 ligands, the anti-inflammatory activity of M2 macrophage is inhibited but without evident changes in cell surface markers (158). On the contrary, inhibition of TLR4 caused a shift from inflammatory M1 macrophages toward M2-dominant macrophages (159), suggesting that TLRs act as a switch that plays a role in the regulation of inflammation. In the downstream of TLRs, interleukin-1 receptor-associated kinase (IRAK) -m has been identified as an inhibitor of TLR signaling (160). One study found that knockdown of IRAK-m promoted M1 polarization and inhibited M2 polarization during the mycobacterium tuberculosis infection (161). But the discovery of IRAK-m-mediated local immunosuppression in periprosthetic tissues hints that macrophages have a self-protective mechanism, wherein the wear debris-mediated stimulation is inhibited to prevent overproduction of NK-κB-dependent proinflammatory cytokines and thus suppressing the deleterious host response in AL (162). However, the drawback is that the induction of IRAK-m overexpression triggered by wear debris also appears to conduce to the inhibition of LPS-induced TLR signaling, which leads to low-level biofilm-associated infection and chronic inflammation (162). Therefore, activation of IRAK-m is somehow affected by the local immune environment.

In addition, M2 polarization is regulated by cytokines in the surrounding, especially IL-10 and IL-4. IL-10 is an important anti-inflammatory cytokine (163). Recent studies have verified that IL-10 treatment significantly reduces iNOS expression and promotes CD163 overexpression, suggesting that IL-10 treatment could reprogram bone marrow-derived macrophages (BMDMs) to an M2 phenotype and regulate the process of M1/M2 polarization (164). *In vivo* experiments revealed that the extra addition of IL-10 partially reversed the inhibitory effect of PE particles on bone ingrowth (165). In the aspect of gene regulation, IL-10 not only decreased the activation of signal transducer and activator of transcription 1 (STAT1), NF-κB p65, and c-Jun N-terminal kinase 1 (JNK1) genes but also increased the expression of STAT3 (164). In addition to IL-10, IL-4 also has the ability to switch the M1 phenotype induced by Ti particles into the M2 phenotype (55, 166). Compared with non-activated macrophages, upon IL-4 stimulation, the shift from M1 to the anti-inflammatory M2 phenotype is more thorough (55).

#### 4.2.3 M2 Macrophage in bone remodeling

Macrophages stimulated by cytokines, such as IL-4 and IL-13, have been confirmed to prevent the OC differentiation and inhibit the function of mature OCs (11, 147). In addition, M2 macrophages may promote OB differentiation by producing cytokines that are critical for osteogenesis, including BMP-2, TGF-β, and IGF-1 (11, 167, 168). Also, by interacting with MSCs, M2 macrophages create an anti-inflammatory environment that is conducive to osseointegration (14). A recent study revealed that preconditioning of murine MSCs with IFN-γ and IL-1β highly significant reduction of CD86 and iNOS protein in macrophages under M1 inducers (LPS + IFN-γ) and diminished TNF-α secretion (169). Additionally, CD86 and iNOS protein expression as well as NO and IL-10 secretion were markedly increased under M2a inducers (IL-4) (169). On the other hand, under the stimulation of IL-4, reduced expression of CD86 and iNOS, as well as increased secretion of nitric oxide (NO) and IL-10 could be discovered in macrophages (169). The secretion of IL-10 could be attributed to the phenotype of M2b macrophages which are generally suggested to be the main subtype of macrophages for inflammatory relief (170). In addition, macrophages stimulated by a conditioned medium from preconditioned MSCs (pre-MSC-CM) may display an overall increased phagocytic capacity (171, 172). Also, M2a macrophages are found to undergo reprogramming to an M2b/M2c phenotype after treatment with the pre-MSC-CM (171, 172), indicating an influence of MSCs behavior on the induction of macrophage polarization.

## 5 Regulatory mechanisms of macrophages in AL

**Figure 2:**
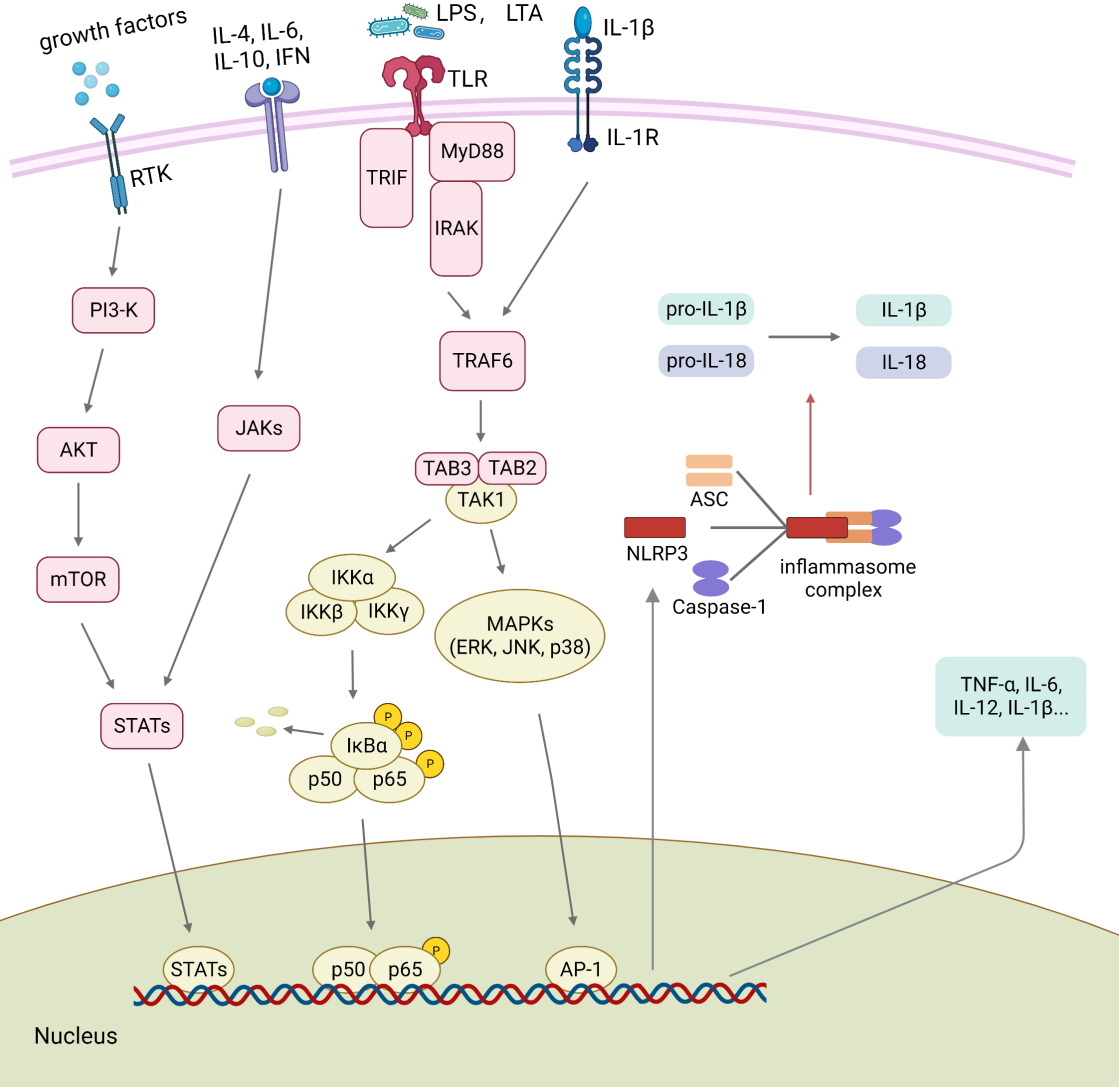
Polarization-related signaling transduction in macrophages. The binding of growth factors or cytokines to their receptors activates STATs through JAKs or PI3K/AKT pathway signaling. Activated TLRs and IL-1βR trigger downstream signaling, including the NF-κB and the MAPK signaling pathways. Several transcription factors, such as AP-1, STATs, and p65, are regulated by the upstream signaling pathways and further promote the assembly of NLRP3 inflammasome. Then, the activated NLRP3 inflammasome promotes the maturation of pro-IL-1β and pro-IL-18. ASC, apoptosis-associated speck-like protein containing a CARD; IFN, interferon; IL, interleukin; LPS, lipopolysaccharide; IRAK, Interleukin-1 receptor-associated kinase; LTA, lipoteichoic acid; MyD88, myeloid differentiation primary response protein 88; NLRP3 NOD-like receptor family pyrin domain containing 3; PAMPs, pathogen-associated molecular patterns; RTK, Receptor Tyrosine Kinases; STATs, signal transducers and activators of transcription; TLR, toll-like receptor; TNF-α, tumor necrosis factor-alpha; TRIF, TIR-domain-containing adapter-inducing interferon-beta.

Several signaling pathways are involved in the process of macrophage polarization (8, 173–179). Various ligands, receptors, transcription factors, and other factors cooperate closely to ensure the precise regulatory capacity of macrophages (8). New research has found that several cytokines can influence M1/M2 polarization through NF-κB, MAPK, and JAK/STAT pathways which are involved in AL (55, 166, 180). Furthermore, depending on the amounts and ratios, molecules in the microenvironment can antagonize or synergistically act, thus in favor of certain macrophage phenotypes (181). Some regulatory mechanisms related to macrophage polarization in the microenvironment of the bone-implant interface are described below (**Figure 2**).

### 5.1 Toll-like receptors

#### 5.1.1 Overview of toll-like receptors

TLRs have been intensively studied in innate immunity regarding the recognition of PAMPs (182). It has been reported that TLR2 or TLR4 can recruit MyD88 and further bind to the IRAK to form a signal complex called Myddosome. The Myddosome-complex recruits the ubiquitin ligase TNF receptor-associated factor 6 (TRAF6), which triggers the TAK1 kinase signaling cascade pathway and ultimately leads to NF-κB nuclear translocation through the phosphorylation and activation of IκB kinase α/β (IKKα/β) (63, 183, 184).

#### 5.1.2 Toll-like receptors in macrophage polarization

TLRs are closely related to macrophage polarization (63). HMGB1 is an important mediator that induces M1 polarization through the activation of absent in melanoma 2 (AIM2) inflammasome, TLR2/4 and RAGE/NF-κB signaling pathways in macrophages (124). In addition, LPS can activate downstream TAK1, NF-κB, and MAPK signals through the TLR4/MyD88 pathway, which increases the expression of cyclooxygenase-2 (COX-2) and iNOS, promotes M1 polarization and leads to the up-regulated expression of cytokines TNF-α, IL-1β, IL-6, and PGE2 (61, 63). The particles with LTA adhering could increase the expression of proinflammatory factors by the activation of TLR2/NF-κB and MAPK pathways (32, 185). Previous studies have shown that a variety of TLRs, such as TLR2, which participate in local inflammatory and immune responses, are present in periprosthetic tissues after the revision of total hip arthroplasty (rTHA) (43).

#### 5.1.3 NLRP3 inflammasome

NLRP3 inflammasome widely exists in macrophages, granulocytes, antigen-presenting cells (APC), and other immune cells. It consists of the NLRP3 (sensor protein), ASC (adapter protein), and caspase-1 (186). The initiation of NLRP3 inflammasome could be induced by multiple inflammatory stimuli, such as PAMPs. As the core protein of the NLRP3 inflammasome complex, NLRP3 senses endogenous DAMPs and microbial ligands. The production of ROS, low level of intracellular potassium (K^+^), and the release of lysosomal protease into the cytoplasm are all upstream mechanisms that trigger the activation of NLRP3 inflammasome in several conditions (187–189). NLRP3 inflammasome which is activated by pathologically stimulated macrophages could regulate the production of IL-18, IL-1β, and cleavage of caspase-1 (186, 188, 189).

On the other hand, studies have also found that NLRP3 inflammasome participates in macrophage polarization (89, 175, 190, 191). In a co-culture experiment, force-pre-treated human periodontal ligament cells (hPDLCs) promote M1 polarization and increase the secretion of IL-1β in macrophages *via* the activation of NLRP3 inflammasome (191). On the contrary, by inhibiting the activation of NLRP3 inflammasome, metformin induces M2 polarization in macrophages and promotes wound healing in rat dorsal skin (175). Moreover, USP19 could directly promote M2 polarization by suppressing the activation of NLRP3 inflammasome to interferon regulatory factor-4 (IRF-4) (89). Bruton’s tyrosine kinase (BTK) is a key factor in TLR4-related pathways, which could activate NF-κB signaling and promote p65 phosphorylation, ASC oligomerization, and caspase-1 activation (192). It has been recently found that BTK could promote TiAl6V4 alloy particles (TiPs)-induced inflammation in BMDMs by positively regulating NF-κB activation, NLRP3 inflammasome formation, and M1 polarization (193). On the contrary, NLRP3 silencing attenuates the promotive effect of conditioned exosomes on M1 polarization in a particles-induced osteolysis model (194).

#### 5.1.4 Syk

Syk is a key component of TLRs in recognizing PAMPs and activating immune responses (195–199). Moreover, Syk plays a critical role in LPS-induced M1 polarization (200). In monocytes, pharmacological inhibition of Syk prevents LPS-induced TLR4 phosphorylation (199), suggesting that Syk may be involved in tyrosine phosphorylation of the TIR domain in TLR4. Recently, PMMA and HA particles were confirmed to augment the expression of CD86, and secretion of cytokines, such as TNF-α and IL-6, in a Syk- and MAPK-dependent (phosphorylation of ERK and p38) manner (56). Also, Syk is reported to be closely associated with the NLRP3 inflammasome-mediated proinflammatory cytokine release (201). In addition, Syk could control NLRP3 activation and IL-1β synthesis in macrophages in response to fungal infections (202).

### 5.2 NF-κB Signaling Pathway

#### 5.2.1 Overview of NF-κB signaling

In the resting state, NF-κB dimers are sequestered in the cytoplasm in an inactive state by the IκB family of proteins, including IκBα, IκBβ, IκBϵ, and the NF-κB precursors, p105 and p100. The IκB kinase (IKK) complex consists of kinases IKKα, IKKβ, and IKKγ. Upon receiving the activation signals, IκB protein is phosphorylated by IKK complexes, leading to proteasomal degradation of IκB. The released NF-κB dimers are then translocating into the nucleus, where they can bind to specific sites on DNA to regulate gene transcription (75).

#### 5.2.2 NF-κB signaling in macrophage polarization

LPS-induced M1 polarization is dependent on NF-κB p65 activation, and the treatment with IKKβ inhibitors reduces the mRNA expression of M1 markers in macrophages (203). Therefore, IKK inhibitors reduce LPS-induced expressions of IL-1, IL-6, IL-10, TNF-α, and IFN (204). Recent studies have revealed that diverse wear particles (e.g., Ti, TiPs, HA, PE, PMMA) could induce the macrophage to release proinflammatory cytokines and chemokines, which are accomplished by the activation of NF-κB signaling pathways and associated macrophage polarization (15, 26, 54, 205). Qiu et al. found that stimulation of LPS or Ti particles could up-regulate p-IKKβ, p-IκBα, and p-p65 and significantly increase the translocation of p65 into the nucleus in BMDMs. Meanwhile, these particles-induced inflammatory infiltrations increase the number of OCs (TRAP-positive cells) and thereby decrease bone mineral density, eventually leading to PPOL (15). Similarly, Gao et al. found that PMMA particles could up-regulate iNOS and decrease the production of Arginase-1 (Arg-1) and IL-10 by the activation of p65 nuclear translocation in macrophages (54).

#### 5.2.3 NF-κB signaling in osteoclastogenesis

Under the same settings, NF-κB can be activated by RANKL or LPS to augment particles-induced bone loss *via* the enhancement of osteoclastogenesis (206, 207). Upstream stimuli-triggered NF-κB signaling could act on transcription factors (c-fos and NFATc1) and regulate the expression of osteoclastogenesis-related genes, thus promoting OC differentiation and functions (206, 208, 209).

### 5.3 MAPK signaling pathway

#### 5.3.1 Overview of MAPK signaling

The MAPK signaling pathway takes part in cell differentiation, proliferation, and apoptosis (210). MAPK pathways are organized into three-tiered cascades consisting of three factors: MAPK, MAPK kinase, and MAPKK kinase. During the phosphorelay process, MAPKKKs which are serine/threonine protein kinases, phosphorylate and activate MAPKKs, and then dually phosphorylate the threonine and tyrosine residues of the conserved TXY motif that belongs to the activation loop of MAPKs, including extracellular signal-regulated kinase (ERK), JNK, and p38 (210, 211).

#### 5.3.2 MAPK signaling in macrophage polarization

MAPK signaling pathway is closely associated with macrophage polarization (61, 212–214). The activation of the MAPK signaling pathway promotes polarization of M1 macrophages, expression of iNOS, and down-regulation of CD206, thereby mediating the secretion of cytokines, such as TNF-α, IL-1β, and IL-6 (214). On the other hand, inhibiting the phosphorylation of JNK, ERK, and p38 in MAPK pathways may switch repolarized M1 to reprogrammed M2 phenotype (213, 214). Studies elucidate that the MAPK signaling pathway is located downstream of TLRs, wherein LPS could activate the MAPK pathway through TLR4/MyD88 pathway to induce M1 polarization and participate in the inflammatory response (61, 62, 215). Further, PMMA and hydroxyapatite (HA) particles are reported to result in M1 polarization by increasing phosphorylation of ERK and p38 (56). Similar to NF-κB signaling, MAPK also mediates wear particles-induced OC differentiation and the following osteolysis (206).

### 5.4 JAK/STAT Signaling Pathway

#### 5.4.1 Overview of JAK/STAT signaling

STAT proteins are potent cytoplasmic transcription factors wherein several family members have participated in macrophage polarization and OC formation, including STAT1, STAT3, and STAT6 (216, 217). Growing studies reveal the involvement of the JAK/STAT pathway in regulating multiple biological events, such as innate and adaptive immunity, cell growth and differentiation, and programmed cell death (217). In the aspect of functioning, phosphorylated STATs promote monomeric dimerization through their SH2 domains and further translocation into the nucleus, where STATs regulate the transcription of target genes (216, 217).

#### 5.4.2 JAK/STAT signaling in macrophage polarization

Several studies have revealed the mediating role of STATs in macrophage polarization. PARP14 silencing or RBM4 knockdown can promote IFN-γ-induced signaling transduction *via* STAT1 activation, which leads to M1 polarization in macrophages (218, 219). Also, STAT1/6 pathway mediates M1/M2 polarization in macrophages after physalin D stimulation (220). By phosphorylating STAT6, protocatechuic acid (PCA) decreases the activation of NF-κB signaling and gives a bias towards M2 polarization over M1 polarization in macrophages (173). Conclusively, an obvious antagonism between STAT1 and STAT6 has been described to promote M1 and M2 cell polarization, respectively (220). In addition, the activation of JAK/STAT3 signaling facilitates macrophage transformation to M2c polarization (180). In an AL mouse model, compared with the pure stimulation of UHMWPE particles, the extra addition of IL-10 significantly decreases iNOS-positive cells and increases CD163-positive cells *via* reducing the transcription of STAT1, NF-κB p65, and JNK1, and promoting the expression of STAT6 (164).

#### 5.4.3 JAK/STAT signaling in osteoclastogenesis

Compared with STAT1 and STAT6, STAT3 is closely related to osteoclastogenesis. Therefore, inhibition of STAT3 can negatively affect RANKL-mediated OC formation and functions (221). An *in vivo* experiment demonstrated that treatment with TiPs pellet could promote the expression of STAT3 and production of RANKL in OBs, thereby stimulating OCs formation in particles-induced osteolysis models, whereas the activation of STAT3 also mediates nano-particles-induced IL-6-dependent inflammatory response in OBs (222).

### 5.5 Calcium (Ca^2+^) signaling

#### 5.5.1 Overview of Ca^2+^ signaling

**Figure 3:**
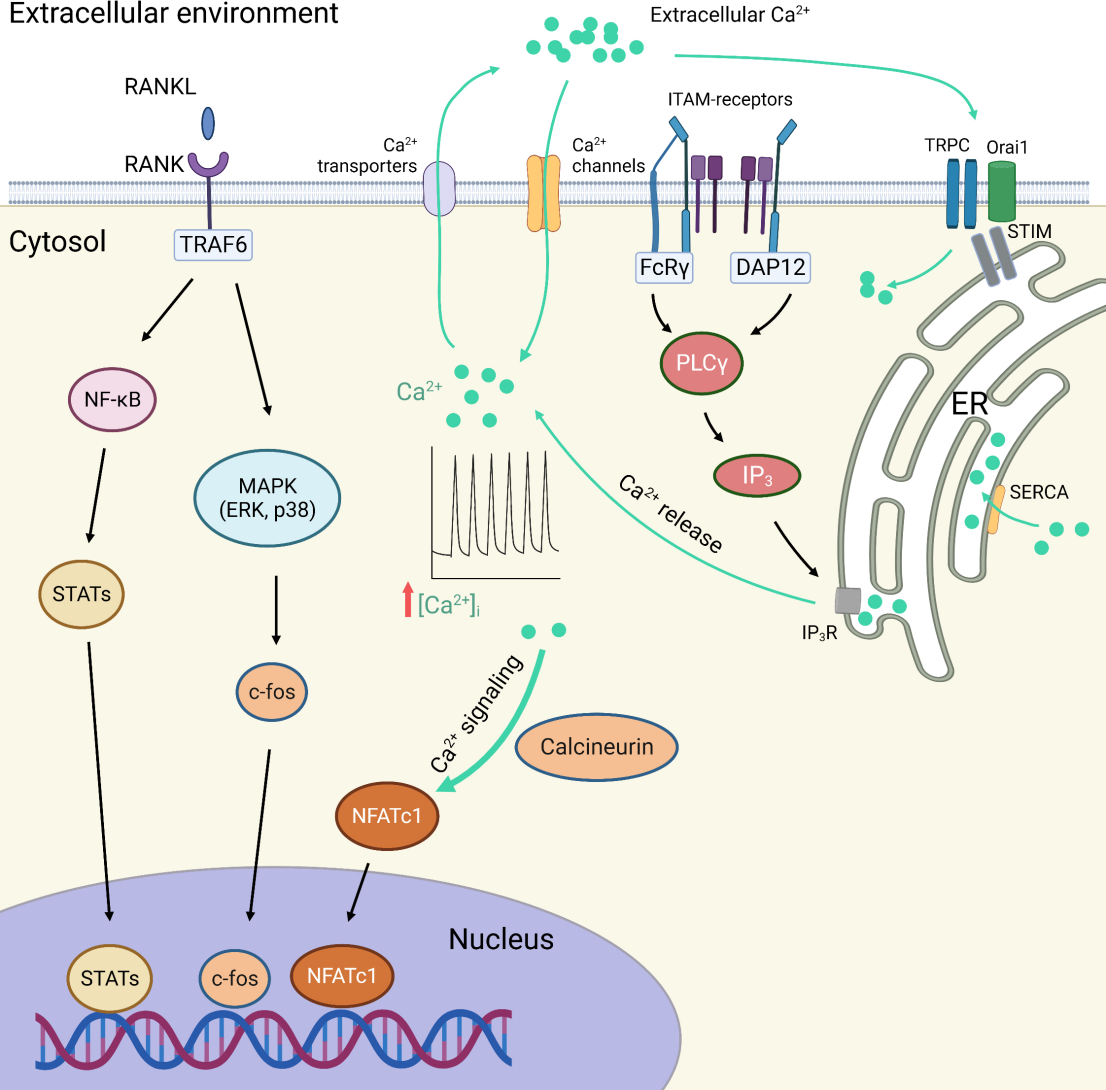
RANKL and calcium (Ca^2+^) signaling in macrophage polarization and osteoclastogenesis. The transcriptional factors responsible for macrophage polarization and osteoclastogenesis are regulated by the NF-κB and MAPK signaling pathways, as well as the Ca^2+^ signaling pathways. The excitability of Ca^2+^ signaling is determined by intracellular Ca^2+^ oscillations, which are attributed by the extracellular Ca^2+^ influx, the effect of ITAM/PLCγ/IP_3_ signaling on the release of Ca^2+^ from ER, and the capacity of collecting Ca^2+^ from the cytosol by SERCA. In addition, depending on the depletion of ER Ca^2+^ storage, extracellular Ca^2+^ entry could also be accomplished by the activation of STIM-mediated TRPC channels and Orai1 channels, which is the so-called SOCE mechanism. Ca^2+^, calcium ions; DAP12, DNAX-activation protein of 12 kDa; ER, endoplasmic reticulum; FcRγ, Fc receptor gamma-chain; IP_3_, inositol 1,4,5-trisphosphate; IP_3_R, inositol 1,4,5-trisphosphate receptor; ITAM, immunoreceptor tyrosine-based activation motif; MAPK, mitogen-activated protein kinases; NFATc1, nuclear factor of activated T cells c1; PLCγ, phospholipase C-gamma; RANK, receptor activator of nuclear factor-kappa B; RANKL, receptor activator of nuclear factor-kappa B ligand; SERCA, Sarco/endoplasmic reticulum Ca(2+)-ATPase; SOCE, store-operated Ca^2+^ entry; STATs, signal transducers and activators of transcription; STIM, stromal interaction molecule; TRAF6, tumor necrosis factor receptor-associated factor 6; TRPC channels, transient receptor potential canonical channels.

The centration of Ca^2+^ in the cytoplasm ([Ca^2+^]c) is 20, 000 times lower than that outside the cell. This is achieved by the Ca^2+^-related transportation and exchange under the dependence on specific proteins. When activated by a variety of external stimuli, cells respond by an increase in the [Ca^2+^]c and trigger downstream signaling, in the form of Ca^2+^ spikes or oscillations (223). Ca^2+^ signaling participates in various biological processes, resulting from a complex switch between the activation and inactivation of Ca^2+^-permeable channels (224). The excitability of Ca^2+^ signaling is determined by intracellular Ca^2+^ oscillations, which are attributed by the extracellular Ca^2+^ influx, the effect of ITAM/PLCγ/IP_3_ signaling on the release of Ca^2+^ from ER, and the capacity of collecting Ca^2+^ from the cytosol by SERCA. In addition, depending on the depletion of ER Ca^2+^ storage, extracellular Ca^2+^ entry could also be accomplished by the activation of STIM-mediated TRPC channels and Orai1 channels, which is the so-called SOCE mechanism (225, 226) (**Figure 3**).

#### 5.5.2 Ca^2+^ signaling in macrophage polarization

Ca^2+^ signaling is related to behavior changes in macrophages, such as polarization and phagocytosis, largely depending on Ca^2+^ uptake in mitochondria (227). Many studies have implicated that distinct Ca^2+^ entry channels determine the IFN-induced M1 polarization or IL-4-induced M2 polarization. Naive or M2 macrophages exhibit a robust Ca^2+^ entry that is dependent on the activity of Orai1 channels (228). As a result, blockade of Ca^2+^ entry inhibits NF-κB/STAT1 or STAT6 signaling events and consequently lowers cytokine production that is essential for M1 or M2 polarization in macrophages (228). In detail, Ca^2+^ influx facilitates M1 polarization, enabling the high productivity of proinflammatory mediators, such as cytokines and chemokines (229). Transient receptor canonical ion channel 1 (TRPC1)-mediated calcium entry seems to play a crucial role in M1 polarization, due to a non-selective TRPC1 current is found in macrophages with M1 phenotype (228, 230). Therefore, knockdown or blockade of the Kir2.1 channel significantly suppresses M1 polarization and promotes M2 polarization (231). Of note, a study found that transient receptor potential vanilloid 1 (TRPV1)-induced Ca^2+^ influx could promote the phosphorylation of Ca^2+^/calmodulin-dependent protein kinase II (CaMKII), but leads to the inhibition of M1 polarization (232), bringing uncertainty in elucidating regulatory mechanism between Ca^2+^ signaling and macrophage polarization.

#### 5.5.3 Ca^2+^ signaling in osteoclastogenesis

Except for macrophage polarization, Ca^2+^ oscillations are the well-known mechanism in triggering RANKL-induced osteoclastogenesis and bone resorption (233, 234). The increase in the [Ca^2+^]c is a fundamental process for mediating OC biology, involving in OC proliferation, differentiation, and resorptive function. At the molecular level, cytosolic Ca^2+^ binds to calmodulin and subsequently activates calcineurin, leading to the activation of NFATc1 which is required for OC differentiation (235). Therefore, by blocking the Ca^2+^ entry channels, inhibition of the activity of Ca^2+^/calmodulin‐dependent protein kinase IV (CaMKIV) and calcineurin will lead to a reduction in the nuclear translocation of c-Fos and NFATc1, ultimately resulting in the suppression of osteoclastogenesis (236). Similarly, directly interfering with intracellular Ca^2+^ oscillations would also have the same negative impact on osteoclastogenesis (237). More content associated with Ca^2+^ oscillations in OC biology has been reviewed in the article by Okada H, et al. (234).

## 6 Future directions

Functional changes of macrophages reflect the intricate and constant regulation of the local environment by the network of cells and cytokines. While macrophage phenotypes have been roughly classified into representative M1 and M2 subtypes, investigating the concrete mechanism for the shift between proinflammatory phenotype and anti-inflammatory state is still challenging, especially in identifying responsible genes and proteins. In this regard, Ca^2+^ signaling based on different stimuli shows bidirectional effects on both macrophage polarization and osteoclast activation, which may be worth further investigation. While it seems clear that the behavior changes of macrophages, such as phagocytosis, pyroptosis, and apoptosis, are closely related to AL pathology, the triggered conditions and signaling events involved have not been fully elucidated. While PAMPs and DAMPs contribute to the response of macrophages to wear particles *via* TLRs, the main reason for the associated activation of TLRs is still controversial. Likewise, DAMPs affect macrophage programming is also unclear. Therefore, the role of PAMPs and DAMPs in AL still needs to be further elucidated. In addition, the local environment in periprosthetic tissue is complex due to the diversity of involved immune cells, such as dendritic cells and lymphocytes, thus highlighting the importance of intercellular communications *via* different signals. To address unresolved issues, applying CRISPR/Cas9 technology which enables accurate and efficient genome editing, would help in effectively elucidating the underlying mechanisms of macrophages in AL (238).

For the therapeutic strategy of AL, we believe the relief of inflammation and inhibition of bone resorption are keys to success. Through an in-depth study into the mechanisms of interaction between cytokines and macrophages, we may instruct a more accurate regulation of the inflammatory process in order to maintain a balance between immune defense and tissue homeostasis.

## Author contributions

YC mainly contributed to the topic determination, literature selection, and draft written. YW (leading), TY, ZZ, JG, and QM screened detailed information from the literature and helped in the manuscript discussion. SS and ZL approved the direction of the topic, revised the manuscript, and supervised the work.
